# EneA of *Aspergillus fumigatus* is a regulator of secondary metabolism and enhances *nscA* expression in presence of polyenes and *Streptomyces*

**DOI:** 10.1038/s41598-026-47215-0

**Published:** 2026-04-09

**Authors:** Oskar Bunz, Jennifer Gerke, Oliver Bader, Emmanouil Bastakis, Merle Aden, Kai Heimel, Ralf Bürgers, Gerhard H. Braus, Christoph Sasse

**Affiliations:** 1 Department of Prosthodontics, University Medical Center Goettingen, Göttingen, Germany; 2 Institute of Organic Chemistry, Leibniz University Hannover, Hannover, Germany; 3 Institute for Medical Microbiology and Virology, University Medical Center Goettingen, Göttingen, Germany; 4 Institute of Microbiology and Genetics, Department of Molecular Microbiology and Genetics and Goettingen Center for Molecular Biosciences (GZMB), University of Goettingen, Göttingen, Germany; 5 Institute of Microbiology and Genetics, Department of Microbial Cell Biology, Göttingen Center for Molecular Biosciences (GZMB), University of Goettingen, Göttingen, Germany

**Keywords:** *A. fumigatus*, Neosartoricin production, Amphotericin B resistance, *Streptomyces noursei*, Secondary metabolism, Biotechnology, Microbiology, Plant sciences

## Abstract

*Aspergillus fumigatus* is a soil born fungus, which can induce severe aspergillosis in immunocompromised patients. In this study, we investigated the zinc cluster transcription factor EneA (*e*nhancer of *nscA* *e*xpression *A*) of *A. fumigatus*, which is required for adaption to polyenes and for competition with soil living streptomycetes. In presence of polyenes, the polyketidesynthase encoding gene *nscA*, which is part of the neosartoricin/fumicycline gene cluster, is induced in dependency of EneA *via* NscR. In contrast, high levels of *eneA* transcripts lead to increased *nscA* expression in the absence of *nscR*, suggesting differing regulatory pathways for neosartoricin production depending on EneA. Moreover, overexpression of *eneA* leads to the induction of genes from nine different secondary metabolite clusters, comprising several of which are known to produce toxins, revealing EneA as a global regulator of secondary metabolism. We demonstrate that these metabolites reduce amphotericin B toxicity and inhibit the growth of streptomycetes. Finally, we show that polyenes and metabolites derived from *Streptomycetes noursei* promote increased neosartoricin production *via* EneA. These findings suggest that the EneA-dependent adaptation of *A. fumigatus* in its natural habitat may contribute to enhanced tolerance to antifungal agents, such as amphotericin B, and augmented resistance to the host’s immune defenses.

**Supplementary Information:**

The online version contains supplementary material available at 10.1038/s41598-026-47215-0.

## Introduction

Fungi compete and communicate with other microorganisms and must protect themselves against fungivors in soil environments. To this end, they synthesize *s*econdary *m*etabolites (SMs) that deter predators or inhibit competitors growth^1–3^. Besides fungal protection, SMs contribute to developmental processes, as shown for *A. nidulans*^4^. SMs are encoded by gene clusters that generally contain genes for a PKS (*p*oly*k*etid*s*ynthase) or an NRPS (*n*on-*r*ibosomal *p*eptid*s*ynthase) as well as a *t*ranscription *f*actor (TF) that controls cluster expression^5–7^. Beyond these internal transcription factors, fungi contains global regulators that are not located within a cluster and can control gene expression of multiple SM clusters^1^.

TFs that regulate secondary metabolites commonly belong to the fungal specific group of *z*inc *c*luster transcription *f*actors (Zcf)^7^. In *A. fumigatus* and *Candida albicans*, this group of regulators is also involved in adaptation to antifungal drugs and thus also in the development of resistance mechanisms^8–11^. *A. fumigatus* represents a global medical problem as an opportunistic pathogenic fungus causing severe aspergillosis with high mortality in immunocompromised patients^12,13^. In addition, the fungus exhibits a substantial capacity to develop resistance mechanisms against antifungal treatments, representing a growing concern that demands attention^13^. Consequently, investigating transcriptional regulators that confer drug resistance is essential to understand how *A. fumigatus* adapts to and survives antifungal treatment.

Widely used antifungal drugs are azoles and polyenes. Azoles inhibit the ergosterol biosynthesis by blocking the Cyp51A enzyme^14,15^, whereas polyenes directly target the membrane by binding at ergosterol^16–18^. Azoles are the first-line treatment, as they are highly effective and favorable side-effect profile. However, a significant disadvantage is the increasing incidence of resistances^19^. Polyenes such as amphotericin B are often reserved as the second-line of treatment, as resistance is much less common here, although these agents are associated with more severe adverse effects^20,21^.

Typical mechanisms of azole resistance involve point mutations combined with tandem repeats in the promoter region of the drug target encoding gene, *cyp51A*^22^. In addition, overexpression of efflux pumps are a common mechanism across multiple fungal pathogens, including *A. fumigatus*^23,24^. These transporters are frequently regulated by fungal specific zinc cluster transcription factors harboring gain of function mutations that trigger overexpression of transporter genes^25–27^. In contrast, resistance to polyenes such as amphotericin B, is comparatively rare. An increased amphotericin B resistance has been shown for *Candida* spp linked to mutations in ergosterol biosynthetic genes^28,29^. Adaptation to *r*eactive *o*xygen *s*pecies (ROS) appears to be an additional important factor for reduced susceptibility to polyenes, as shown for the filamentous fungus *Aspergillus terreus*. This fungus exhibits increased natural resistance to amphotericin B that has been associated with the oxidative stress response^30^. However, recent work demonstrated that overexpression of specific zinc cluster transcription factors increases resistance to amphotericin B independently of an altered ROS response^10^.

In this work, the zinc cluster transcription factor EneA (*e*nhancer of *nscA* *e*xpression *A*) was analyzed, which activates several SM clusters including the neosartoricin/fumicycline gene cluster when overexpressed. These metabolites reduce the effect of amphotericin B and inhibit the growth of the soil bacterium *S. noursei*^31^. The presence of polyenes and/or metabolites from *S. noursei* induce the expression of *eneA*, which in turn upregulates the PKS encoding gene *nscA*. Importantly, NscA-mediated production of neosartoricin/fumicycline is known to suppress the human immune response^32^. Together, our observations suggest that EneA enhances fungal survival in the environment and promotes adaptation within the host *via* the production of SMs.

## Results

### EneA is a global regulator of secondary metabolism in *A. fumigatus*

**Fig. 1:**
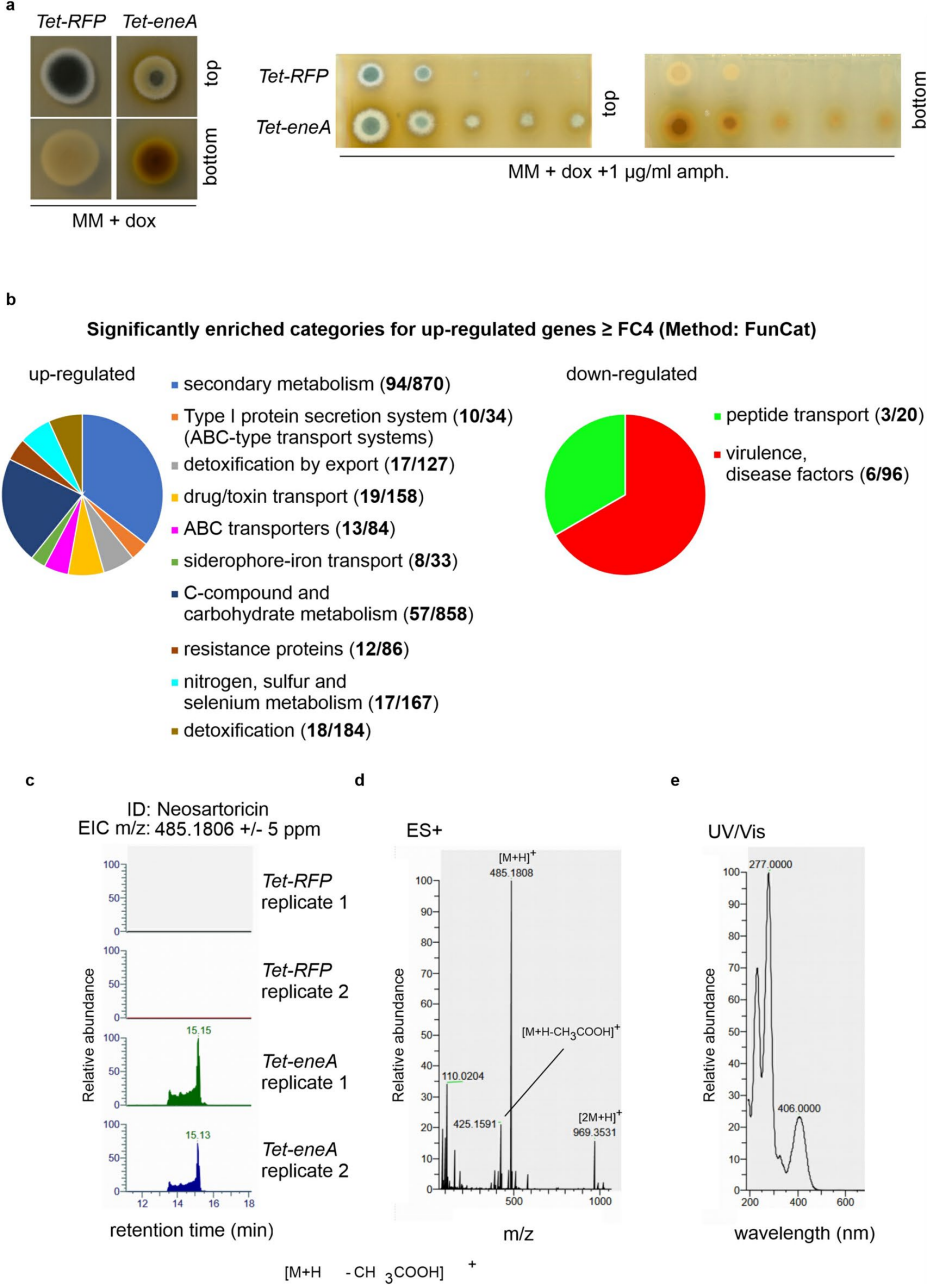
Overexpression of *eneA* induces the expression of secondary metabolite clusters. (**a**) Overexpression of *eneA* using the *TetOn* system in presence of 50 µg/ml doxycycline. As control, a *Tet-RFP* strain was used. Overexpression of *eneA* leads to the production of colorants and increases the resistance to amphotericin B. Categories for up-regulated and down-regulated genes ≥ FC4 using FunCat (p-value ≤ 0.05)^35^. (**b**) Overexpression of *eneA* primarily leads to the up-regulation of genes involved in secondary metabolism. (**c**) EIC (extracted ion chromatogram) of neosartoricin detected by CAD. The metabolite was validated by exact mass (positive mode) (**d**) and UV/Vis (**e**). The metabolite was only identified in the *Tet-eneA* strain but not in the *Tet-RFP* control strain. Extracts from two different plates of each strain were used for LC-MS analysis.

Overexpression of the transcription factor encoding gene *eneA* (*e*nhancer of *nscA* *e*xpression *A*), previously published as *zcf63* and *mdd2*^10,33^, has been shown to increase the resistance to amphotericin B and secretion of colorants into the medium^10^, Fig. 1a). Nevertheless, target genes of *eneA* are so far elusive. RNAseq experiments were carried out using the *Tet-eneA* (*Tet-zcf63*) overexpression strain in presence of doxycycline to identify genes regulated by EneA. The *Tet-RFP* strain AfGB118^34^, served as control (supplementary Table S1-S5). Analyses of the received RNAseq data revealed 474 up-regulated genes with a *f*old *c*hange (FC) ≥ 4 in comparison to the control (supplementary Table S3). In contrast, only 84 genes were downregulated with a FC ≥ 4 (supplementary Table S5). FunCat analysis of the up-regulated genes^35^, supplementary Table S6 and Table S7) identified 10 significantly overrepresented categories in our data set (Fig. 1b). The highest enrichment was found for genes involved in the type I protein secretion system. EneA induced 29% of all known genes of this category. However, the largest number of genes are associated with secondary metabolism. In total 94 SMs related genes were up-regulated in the *Tet-eneA* strain in comparison to the control which corresponds to 11% of all SM genes in *A. fumigatus*. In contrast to the ten categories of the up-regulated genes, only two significantly enriched categories (transport mechanism and virulence) for the down-regulated genes were identified (Fig. 1b, right panel).

A more detailed analysis of the data revealed that eleven putative zinc cluster transcription factors encoding genes in addition to *eneA* were induced with a FC ≥ 4 (supplementary Table S3). Two of them (*nscR* and *ftpR*) are part of gene clusters involved in SM production^36^. Two genes (AFUA_5G14290 and AFUA_4G01470) have been described to be regulated in a fusion/fission mitochondrial mutant strain^33^, one of which (AFUA_5G14290) influences the development of *A. fumigatus* and the other leads to an increased sensitivity to voriconazole^10,33^. Of the remaining six candidates, only AFUA_3G05760 produced a detectable overexpression phenotype, whereas four candidates showed no phenotype^10^ and AFUA_4G01105 has not yet been characterized. The negative regulators NctA and NctB, previously implicated in amphotericin B resistance^11^ are not regulated (*nctA*) or not expressed (*nctB*) in our data set.

Moreover, several stress-response genes were induced, including glutathione-S-transferases (AFUA_2G17300; AFUA_7G05500, and AFUA_3G10830) and copper transporters (AFUA_2G03730, AFUA_3G13660, and AFUA_6G02810) (supplementary Table S5).

Within the category of secondary metabolism, genes from nine different clusters were significantly induced (FC ≥ 4) in the overexpression strain (Table 1, supplementary Table S5). Six of these clusters are already known and characterized^37,38^, including genes connected to the helvolic acid, xanthocillin, as well as the neosartoricin/fumicycline clusters. Moreover, genes from two additional uncharacterized clusters and the partially characterized *ftpR* cluster^36,39^ were induced. Further analysis of SM extracts of the *Tet-eneA* strain *via* LC-MS showed the increased production of metabolites compared to the control (supplementary Fig. S1). One of the increased produced metabolites was identified as neosartoricin by exact mass and UV/Vis (Fig. 1c-e) based on the data of Chooi et al.^32^.

**Table 1:** Up-regulated genes in the *Tet*-*eneA* strain involved in secondary metabolism.

GeneID	Gene	SM cluster	log2(FC) up-regulation
AFUA_3G13660	*crmD*	Fumivaline and Fumicicolin Cluster	5.566259035
AFUA_3G13670	*crmC*	Fumivaline and Fumicicolin Cluster	8.157643354
AFUA_3G13680	*crmB*	Fumivaline and Fumicicolin Cluster	6.561955668
AFUA_3G13690	*crmA*	Fumivaline and Fumicicolin Cluster	9.922057973
AFUA_3G13700		Fumivaline and Fumicicolin Cluster	9.098543529
AFUA_4G14770	*helA*	Helvolic acid (hel) Cluster	5.689664368
AFUA_4G14780	*helB1*	Helvolic acid (hel) Cluster	4.639737327
AFUA_4G14790	*helB2*	Helvolic acid (hel) Cluster	4.793557909
AFUA_4G14800	*helC*	Helvolic acid (hel) Cluster	6.916268917
AFUA_4G14810	*helB3*	Helvolic acid (hel) Cluster	6.651647539
AFUA_4G14820	*helD1*	Helvolic acid (hel) Cluster	6.169614502
AFUA_4G14830	*helB4*	Helvolic acid (hel) Cluster	7.28308375
AFUA_4G14840	*helD2*	Helvolic acid (hel) Cluster	8.056059172
AFUA_4G14850	*helE*	Helvolic acid (hel) Cluster	5.142108573
AFUA_5G02620	*xanG*	Xanthocillin (xan) Cluster	11.1566772
AFUA_5G02630	*xanF*	Xanthocillin (xan) Cluster	12.59946068
AFUA_5G02640	*xanE*	Xanthocillin (xan) Cluster	12.30849184
AFUA_5G02650	*xanD*	Xanthocillin (xan) Cluster	2.268586428
AFUA_5G02655	*xanC*	Xanthocillin (xan) Cluster	4.652967705
AFUA_5G02660	*xanB*	Xanthocillin (xan) Cluster	11.8672004
AFUA_5G02670	*xanA*	Xanthocillin (xan) Cluster	10.04056163
AFUA_7G00120	*nscB/fccB*	Neosartoricin (nsc)/Fumicycline (fcc) Cluster	13.03252568
AFUA_7G00130	*nscR/fccR*	Neosartoricin (nsc)/Fumicycline (fcc) Cluster	10.52571429
AFUA_7G00150	*nscC/fccC*	Neosartoricin (nsc)/Fumicycline (fcc) Cluster	5.60351766
AFUA_7G00160	*nscA/fccA*	Neosartoricin (nsc)/Fumicycline (fcc) Cluster	14.24646328
AFUA_7G00170	*nscD/fccD*	Neosartoricin (nsc)/Fumicycline (fcc) Cluster	14.3131411
AFUA_7G00180	*nscE/fccE*	Neosartoricin (nsc)/Fumicycline (fcc) Cluster	10.89888494
AFUA_8G00370	*fmaB*	Fumagillin (fma) Cluster	2.305584682
AFUA_8G02390	*spyD*	Satorypyrone (spy) Cluster	2.104333593
AFUA_3G01400		Uncharacterized Cluster	6.645322338
AFUA_3G01410		Uncharacterized Cluster	2.965528943
AFUA_5G12700		Uncharacterized Cluster	3.071897337
AFUA_5G12770		Uncharacterized Cluster	4.79814454
AFUA_3G15250		Partially characterized Cluster	7.05261923
AFUA_3G15270		Partially characterized Cluster	4.980130664
AFUA_3G15280		Partially characterized Cluster	4.671092068
AFUA_3G15290	*ftpR*	Partially characterized Cluster	8.672961205

A previous proteomic study profiled *A. fumigatus* cultured with and without amphotericin B supplementation^40^. Comparison of these data with our RNAseq dataset revealed 72 overlapping genes (63 upregulated and nine down-regulated) (supplementary Table S8-9). Of the 63 induced genes, 44 showed an FC ≥ 4, and of the nine suppressed genes, two showed an FC ≥ 4 in our RNA-seq analysis. Nine proteome candidates could not be found in the RNAseq data set. Within the up-regulated genes *rtaA* was identified, encoding a member of the RTA-like protein family which was previously shown to be involved in amphotericin B resistance^40^. With respect to secondary metabolism, only genes/proteins from the neosartoricin/fumicycline and the xanthocillin cluster were represented in both datasets. Notably, EneA itself was not detectable in the proteomics approach of Abou-Kandil et al.^40^.

### EneA is required for the adaption to polyenes and enhances *nscA* expression in presence of nystatin and amphotericin B

**Fig. 2:**
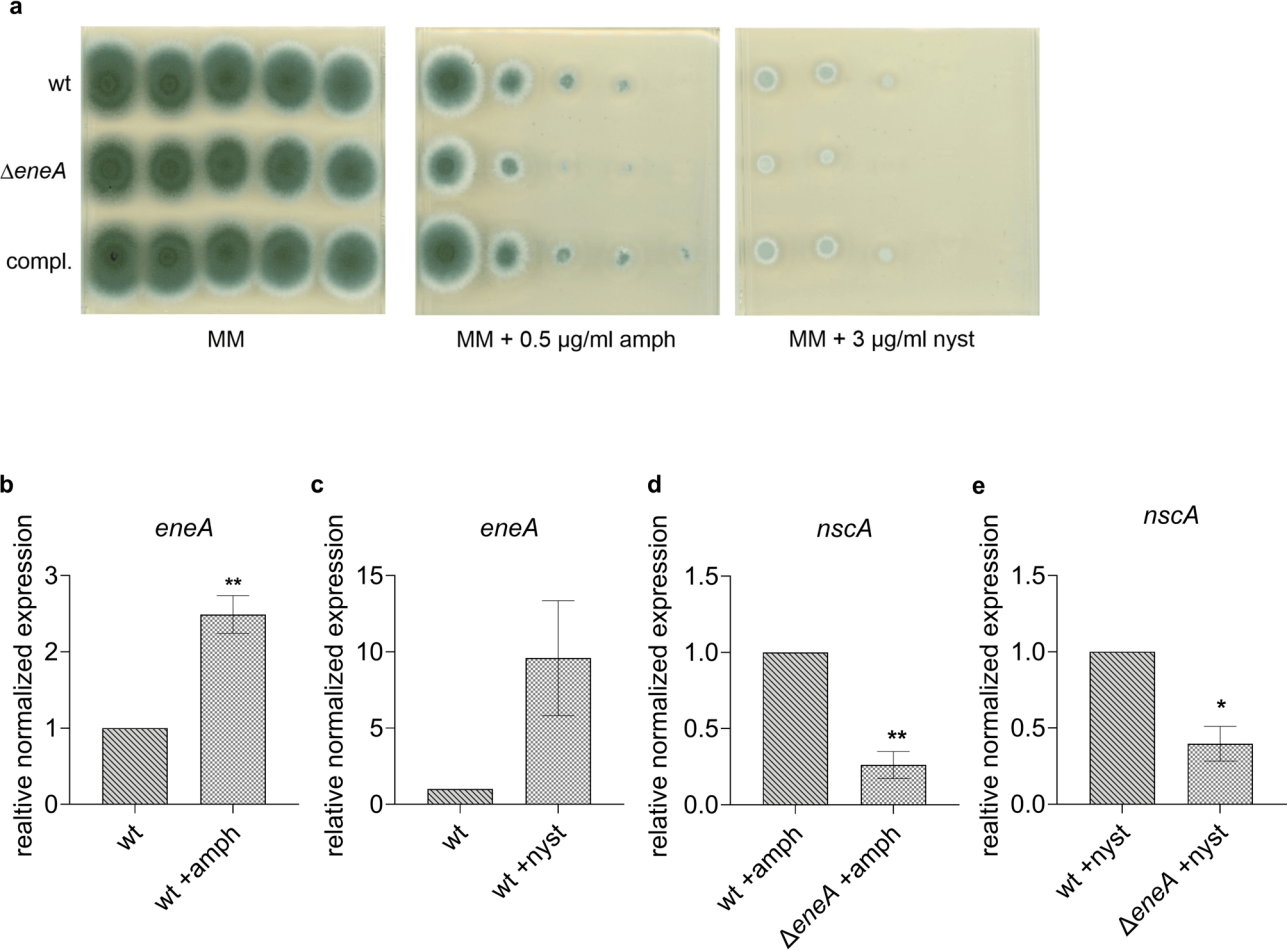
*A fumigatus* EneA is required for polyene resistance and regulates *nscA*. (**a**) Dilution spot-tests of the wild type (wt), the *eneA* deletion strain (Δ*eneA*) and the complementation strain (compl.) on minimal medium (MM) in presence and absence of nystatin and amphotericin B. All strains were spotted in dilution steps starting with 1.5 × 10^5^ spores. Plates were incubated for 3 days at 37 °C. The *eneA* deletion strain shows an increased susceptibility on amphotericin B and nystatin containing medium. (**b**-**e**) qPCR experiments of the wt and the deletion strain in presence and absence of amphotericin B (final conc. 0.65 µg/ml) and nystatin (final conc. 3 µg/ml). Gene expression of *eneA* (**b-c**) and *nscA* (**d-e**) in presence of polyenes were analyzed. Three biological replicates were performed. For normalization, the genes *H2A* and *eIF2B* were used. Error bars represent the *s*tandard *e*rror of the *m*ean (SEM). Significance is shown by asterisks. * ≙ p-value ≤ 0.05, ** ≙ p-value ≤ 0.005. Significances refer to the wt without drug (*ene*A expression) and for the wt with drug (*nscA* expression). *eneA* and *nscA* are induced in presence of both drugs. No expression could be observed for *nscA* in the wt and the Δ*eneA* strain without drugs. Statistical analysis was carried using a student’s t-test.

In order to ascertain whether loss of *eneA* results in increased sensitivity to amphotericin B and whether this is also the case in the presence of other polyenes an *eneA* deletion strain was constructed. The received strain was tested for susceptibility in presence of the polyenes amphotericin B and nystatin in comparison to the wild type. Absence of *eneA* reduces the growth of the fungus in presence of both antifungal drugs. This effect could be complemented *via* reintegration of the gene in the mutant strain (Fig. 2a). In contrast, no difference could be found on minimal medium without drug in comparison to the wild type, indicating that the observed sensitivity of the mutant to these antifungal agents is not due to a general growth defect. Comparison of the *Tet-eneA* RNAseq data set with previously published proteomic results^40^ revealed an overlap of 72 genes, including genes from the neosartoricin/fumicycline gene cluster, indicating a central role of *eneA* in the response to amphotericin B and neosartoricin production when overexpressed. To determine whether the activation of the neosartoricin/fumicycline gene cluster by amphotericin B is dependent on EneA, qPCR experiments were performed (Fig. 2b-e). Therefore, the wild type and the *eneA* deletion were analysed in presence and absence of nystatin and amphotericin B. An increased expression of *eneA* was observed in presence of the used drugs, but only for amphotericin B a statistically significant effect was observed (Fig. 2b-c). Moreover, both polyenes significantly induced the PKS encoding gene *nscA* in the wild type in comparison to the deletion strain. Hence, EneA is required for the adaption to polyenes and acts as enhancer of *nscA* expression in presence this group of antifungal drugs.

### EneA requires *nscR* to induce *nscA* in presence of amphotericin B

**Fig. 3:**
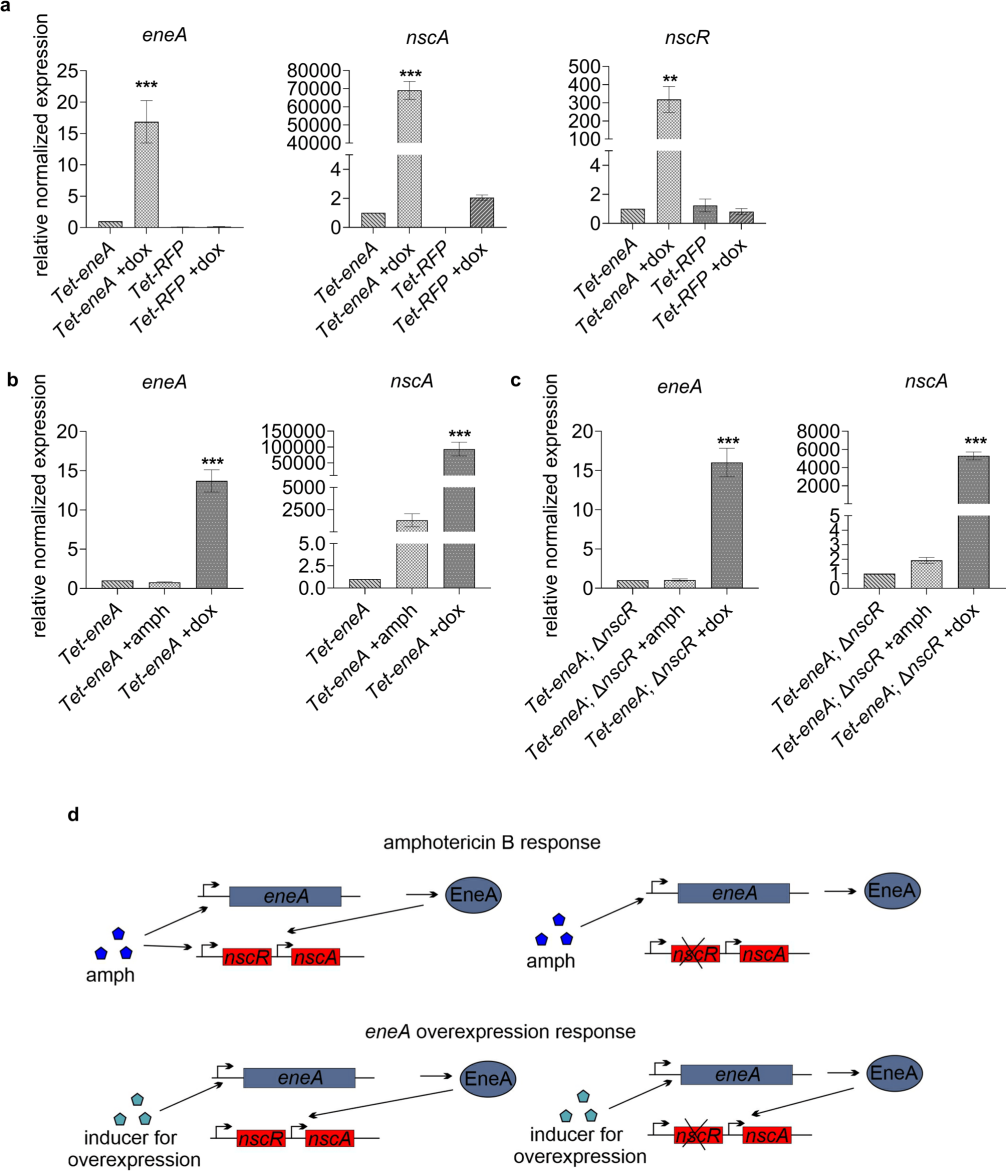
Regulation of *nscA* in dependency of *nscR* and *eneA*. (**a**) qPCR of the *Tet-eneA* overexpression strain in presence and absence of 50 µg/ml doxycycline (dox). As control the *Tet-RFP* strain with and without doxycycline was used. Three biological replicates were carried out. Overexpression of *eneA* induces significantly the expression of *nscA* and *nscR*. No expression of *nscA* could be measured in the *Tet-RFP* strain without doxycycline. (**b-c**) qPCR of the *Tet-eneA* strain and the Tet-eneA; Δ*nscR* strain in presence of amphotericin B (+ amph) with a final conc. of 0.65 µg/ml, doxycycline (+ dox) in a final conc. of 50 µg/ml and without any drug. Four biological replicates were performed. Induction of *nscA* in presence of amphotericin B requires *nscR*. In contrast, *nscA* induction can be induced without *nscR* by overexpression *eneA*. For normalization, the genes *H2A* and *eIF2B* were used for all qPCR experiments. Error bars represent the *s*tandard *e*rror of the *m*ean (SEM). One-way ANOVA was carried out for statistical analysis. Significance is shown by asterisks. ** ≙ p-value ≤ 0.005, *** ≙ p-value ≤ 0.0005. Significances refer to the *Tet-eneA* strain without doxycycline. (**d**) Schematic representation of *nscA* regulation depending on *nscR* and *eneA* in the presence of amphotericin B or upon overexpression of *eneA*.

In presence of polyenes, *eneA* is induced and leads to increased expression of the PKS encoding gene *nscA* (Fig. 2). *nscA* is controlled by the transcriptional regulator *nscR* which is part of the neosartoricin/fumicycline gene cluster^32,36^. Therefore, the dependency between *eneA* and *nscR* or the existence of two independent regulatory mechanisms for the neosartoricin/fumicycline cluster, was investigated. The *Tet-eneA* overexpression strain and a *Tet-RFP* control strain were used to analyze expression of *nscR* and *nscA* under inducing and non-inducing conditions by qPCR (Fig. 3a). In presence of doxycycline, *eneA* expression is significantly induced by more than 15-fold in comparison non-induced sample. The increased expression results in the upregulation of both *nscA* and *nscR* and is consistent with our RNAseq data. In contrast, the transcript level was diminished in the *Tet-RFP* strain in presence and absence of doxycycline in comparison to the *Tet-eneA* control. Nevertheless, this reduced expression of *eneA* does not result in a reduced induction of *nscA* in comparison to the *Tet-eneA* strain. This suggests that increased transcript levels of *eneA* in the *Tet-eneA* strain are not sufficiently high to promote activation of the neosartoricin/fumicycline cluster. Furthermore, the presence of doxycycline itself leads to the induction of *nscA* (increased twofold compared to the non-induced *Tet-eneA* strain), while *nscR* expression is not influenced. However, this is most likely just background noise, as only very weak expression was measured compared to the expression of *nscA* in *Tet-eneA* with doxycycline.

To get a better understanding of the regulatory interaction between *eneA* and *nscR*, *nscR* was deleted in the *Tet-eneA* strain. Subsequently, qPCR was performed with the *Tet-eneA* strain and the *Tet-eneA*; Δ*nscR* strain in minimal medium, in minimal medium with amphotericin B, and in minimal medium with doxycycline (Fig. 3b-c). In presence of doxycycline, but not amphotericin B, *eneA* expression is induced in both strains relative to the non-induced conditions. Moreover, overexpression of *eneA* leads to the activation of *nscA* in in the *Tet-eneA* as well as *Tet-eneA*; Δ*nscR* strain. This shows that once a certain threshold amount of *eneA* transcript is reached, the induction of *nscA* can occur even without the regulator NscR. In contrast, the presence of amphotericin B only weakly induced *nscA* (approx. 2-fold increased) in the absence of *nscR*. Thus, there are two different regulatory pathways for *nscA* (Fig. 3d). One is polyene-induced, whereby the induction of *nscA* by EneA is dependent on *nscR* expression, and the other is *eneA* overexpression-dependent, which does not require *nscR* (Fig. 2b and d, and Fig. 3b-d).

### Supernatant of a *S. noursei* submerged culture induces the expression of *eneA* and *nscA* in *A. fumigatus*

**Fig. 4:**
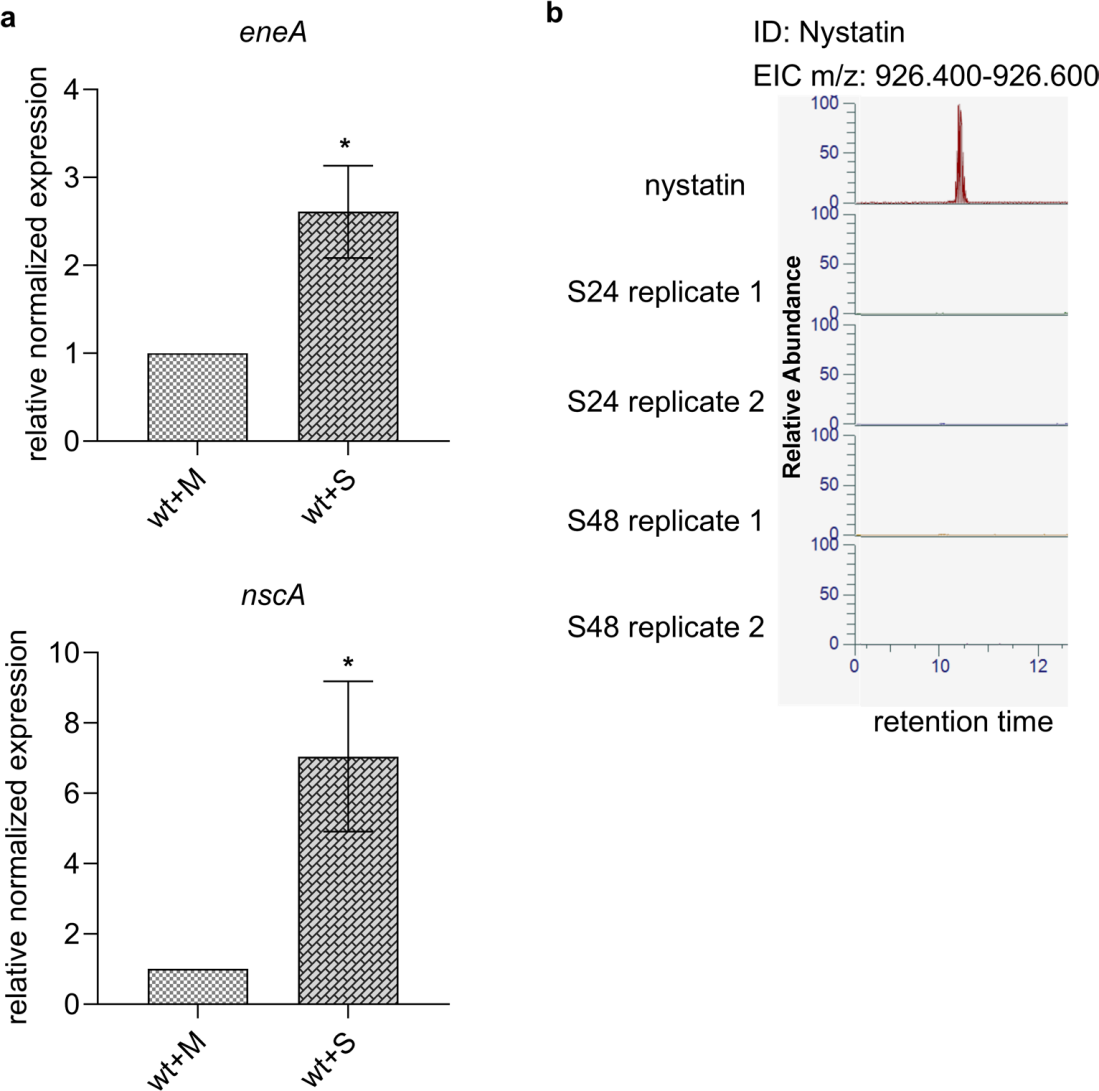
Analysis of co-cultures of *A. fumigatus* and *S. noursei* by qPCR. (**a**) qPCR of the *A. fumigatus* wild type in co-culture with supernatant of a *S. noursei* overnight culture (wt + S). *A. fumigatus* overnight culture plus fresh streptomycete medium without *S. noursei* (wt + M) was used as control. Four biological replicates were carried out. For normalization, the genes *H2A* and *eIF2B* were used. Error bars represent the *s*tandard *e*rror of the *m*ean (SEM). The co-cultivation of *A. fumigatus* and *S. noursei* supernatant leads to the induction of *eneA* and *nscA*. A student’s t-test was carried out for statistical analysis. Significance is shown by asterisks. * ≙ p-value ≤ 0.05. (**b**) EIC (extracted ion chromatogram) of nystatin detected by CAD. This metabolite was validated by exact mass (positive mode) received from the reference of pure nystatin. Nystatin could not be detected in one of the samples of the submerged cultures of *S. noursei* after 24 h (S24h) and 48 h (S48h) incubation at 30 °C in GYM medium. Two replicates were measured.

Polyenes are produced and secreted by soil-dwelling *Streptomyces* spp., which are competitors of *A. fumigatus*^41–43^. König et al. have previously shown that the neosartoricin/fumicycline cluster is activated in presence of *Streptomyces rapamycinicus*^44^. To test whether activation of the neosartoricin cluster is also induced without direct contact with *Streptomyces* in dependency of EneA, *A. fumigatus* was incubated with the supernatant from an overnight culture of *S. noursei*, a nystatin producing streptomycete^31^. The supernatant received from the *S. noursei* significantly induced expression of *eneA* and *nscA* in comparison to the control (Fig. 4). This raises the question whether the induction is polyene-dependent or whether other metabolites in the supernatant of *S. noursei* induce the expression of *eneA*. To this end, secondary metabolites from submerged cultures of *S. noursei* were extracted (supplementary Fig. S2). The samples were analyzed *via* LC-MS for nystatin. As standard, 1 µg of nystatin was used. No nystatin could be detected in any of the tested samples except the control. Consequently, the induction of *eneA* by *S. noursei* is not based on the presence of nystatin, but on other metabolites secreted by this bacterium.

### Overexpression of *ene*A induces SMs involved in polyene resistance and growth inhibition of *S. noursei*

**Fig. 5:**
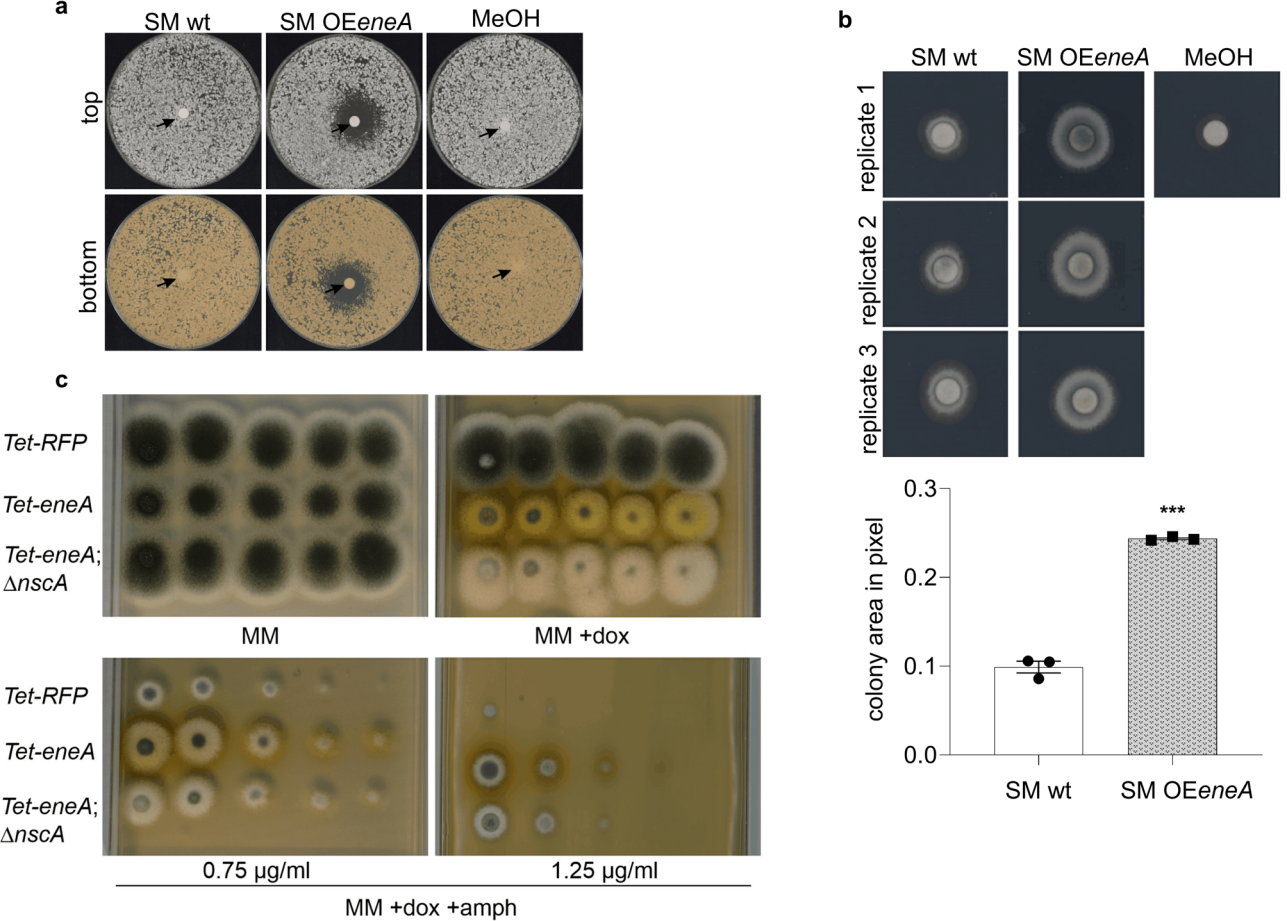
Secondary metabolites from the *A. fumigatus eneA* overexpression strain neutralize amphotericin B and inhibit the growth of the soil bacterium *S. noursei*. (**a**) Disc diffusion assays using extracts of the *OEeneA* strain and the wild type. *S. noursei* was inoculated on GMX plates in presence of fungal extracts (overexpression strain, wild type). Black arrows indicate the paper discs soaked with the two different extracts. As control a paper disc with methanol was used. Growth inhibition is indicated by a halo around the apper discs. (**b**) Plates containing embedded spores of the *(A) fumigatus* wild type and 1.5 µg/ml amphotericin B. In the middle of the plate, a paper disc was added which was soaked with extracts containing secondary metabolites (SM) from the *eneA* overexpression strain or from the wild type. As control, a paper disc containing methanol was used. Plates were incubated for three days at 37 °C. The growth area of the plates were determined and statistical analyzed using student’s t-test with *n* = 3. *** ≙ p-value ≤ 0.0005. Circles and squares represent the individual values of the replicates. Only the extract received from the overexpression strain increases significantly the growth of the fungus in presence of amphotericin B (**b**) and inhibits the growth of the bacterium indicated by a growth halo around the paper disc (**a**). The figure shows exemplary plates. All plates are shown in supplementary Fig. S3. Dilution spot test of the *Tet-prrA* strain and the *Tet-prrA*; Δ*nscA* strain. As reference, the *Tet-RFP* strain was used. Strains were spotted on minimnal medium (MM) minimal medium doxycycline (+ dox) or minimal medium with amphotericin B and doxycycline (+ dox +amph). Amphotericin B was used in a final concentration of 0.75 mg/ml and 1.25 µg/ml. Doxcycline was used in a final concentration of 50 µg/ml. Plates were incubated for three days at 37 °C (**c**). The absence of *nscA* does not increase the susceptibility to amphotericin B.

Overexpression of *eneA* induces the production several different SMs. We thus asked whether the *eneA* dependent secondary metabolites are involved in the observed polyene resistance and in protection against fungal competitors. To activate the different SM clusters an *eneA* overexpression strain was used. The *Tet*-*eneA* overexpression strain required doxycycline for induction that acts as antibiotic preventing experiments with bacteria. As a result, an *eneA* overexpression strain was constructed which contains a copy of the *eneA* gene under the nitrate inducible *niiA* promoter instead of the *TetOn* system. In a disc diffusion experiment, the inhibition of SMs from the OE*eneA* and from the wild type to *S. noursei* was tested (Fig. 5a, supplementary Fig. S3a). The wild type extract showed no effect on the growth of *S. noursei*. In contrast, metabolites from the overexpression strain strongly inhibited growth as reflected by a halo around the paper disc.

To analyze the effect of secondary metabolites on amphotericin B resistance a disc diffusion assay using the same extracts as mentioned above was performed using plates containing wild type spores and amphotericin B (Fig. 5b, supplementary Fig. S3b). As control methanol was used. The extracts of the overexpression strain significantly improved growth in the presence of amphotericin B compared to those derived from the wild type strain. No growth at all was observed on the methanol control plate. These results corroborate that SMs received from an *eneA* overexpression strain suppress the toxic effect of amphotericin B and inhibits the growth of *S. noursei*.

To determine whether neosartoricin is responsible for the reduced susceptibility to amphotericin B, we deleted *nscA* in the inducible *Tet-eneA* overexpression strain and performed dilution spot tests of the *Tet-eneA*, the *Tet-eneA*; ∆*nscA*, and the *Tet-RFP* strain as control (Fig. 5c). Strains were spotted on minimal medium, minimal medium with doxycycline to induce the expression of *eneA* and on minimal medium with doxycycline and amphotericin B. The absence of *nscA* led to a reduced yellow colorant under inducing conditions but did not affect growth in presence of amphotericin B in comparison to the *Tet-eneA* strain. Hence, the observed effect of the SM extracts on amphotericin B does not depend on neosartoricin.

## Discussion

In this study we demonstrate that EneA is part of a polyene response and adaption system of *A. fumigatus*. In presence of polyenes (amphotericin B and nystatin), the expression of *nscA* is induced in dependency of EneA. Furthermore, the presence of amphotericin B but not nystatin induces significantly the expression of *eneA* as well. However, increased expression of *eneA* was already observed in the *Tet-eneA* strain in comparison to the *Tet-RFP* control strain under non-inducing conditions (Fig. 3). This increased basal expression does not lead to increased expression of *nscA* and *nscR*. As a result, a certain threshold expression value of *eneA* is required to regulate target genes. Thus, the observed transcriptional regulation in presence of polyenes seems to have only a subordinate role in the activation of *eneA*. Posttranslational modifications, like phosphorylation, or the interaction with other proteins appears to be more important for the activation of EneA. One possible mechanism could be related to the mode of action of polyenes. This group of antifungals induce perforations in cell membranes, which finally causing oxidative stress in the cell^16–18^. The increased amount of ROS could drive posttranslational modification of EneA like phosphorylation. Beside these modifications, this activation can also be regulated by interaction with other proteins depending on the ROS level. Future works will be therefore interesting to find out how EneA is modified and which are its interaction partners in presence of different polyenes.

Consistent with this, we were able to show that EneA requires *nscR*/NscR to induce *nscA* in the presence of amphotericin B. This data suggests a model in which amphotericin B induces/modifies *eneA*/EneA on transcriptional and posttranslational level, which in turn activates *nscA* in an NscR-dependent manner (*eneA* being upstream of *nscR*; Fig. 3d). In contrast, strong *eneA* overexpression can drive *nscA* expression even in absence of *nscR*, revealing dual regulation of the neosartoricin/fumicycline cluster: *nscR* dependent under amphotericin B exposure and *nscR* independent by increased *eneA* expression. The extent to which a direct protein-protein interaction between EneA and NscR is also required remains unclear. Thus, *A. fumigatus* has the ability to alter the regulation pathway of EneA by changing its gene expression level and by putative posttranslational modifications. Whether this mechanism takes place in the soil or in the host, and whether individual factors or a combination of several components are required, needs further investigations.

In this paper we could show that polyenes lead to the induction of the neosartoricin/fumicycline cluster which confirms the results of Abou-Kandil et al.^40^. Thus, EneA is required for its full activation in presence of polyenes. Moreover, the overexpression of *eneA* leads to increased expression of several other secondary metabolite clusters like the xanthocillin or helvolic acid cluster. However, based on our data, it is not possible to determine the extent to which this occurs *via* direct or indirect regulation. Future ChIP-seq experiments would therefore help to improve our understanding of how EneA regulates secondary metabolite clusters.

The inhibitory effect on the observed bacterial growth can be attributed to individual metabolites like fumivaline, fumicicolin, helvolic acid and neosartoricin/fumicycline for which it has partially been shown that these are toxic to other organisms^44–47^. However, EneA does not activate the fumigermin cluster, which has previously been shown to be induced in the presence of *Streptomyces rapamycinicus*^41^. This suggests that the neosartoricin cluster is activated by a factor that is not species-specific.

We could show that the metabolites received from the *eneA* overexpression strain have the capacity to reduce the toxicity of amphotericin B (Fig. 5b). However, the investigation revealed that neosartoricin itself exerts no influence on the increased adaptation to amphotericin B (Fig. 5c). Consequently, it can be deduced that the observed effect must be attributable to the presence of other metabolites. In order to ascertain the extent to which this phenomenon can be attributed to individual metabolites or to the interaction of several metabolites, future research is required.

**Fig. 6:**
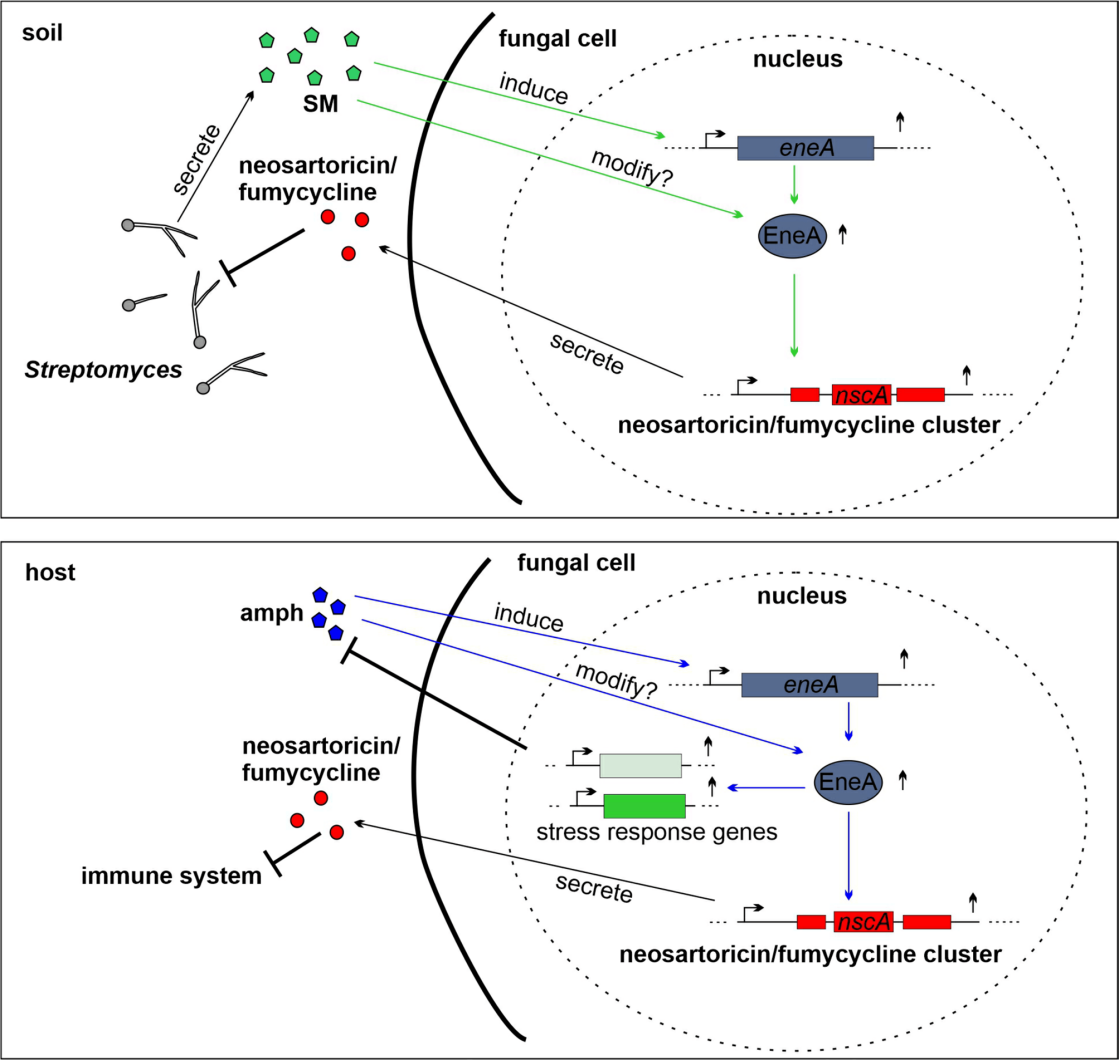
Fungal communication and interaction between *A. fumigatus* and bacteria of the genus *Streptomyces* in the soil. EneA is a key regulator to enforce soil-living streptomycetes and to adapt during polyene treatment in the host. Within the soil *A. fumigatus* has to deal with other microorganism like streptomycetes (upper panel). In the presence of metabolites released from streptomycetes, *eneA*/EneA is activated, leading to increased expression of the neosartoricin/fumicyclin gene cluster. Within the host, this reaction system enables *A. fumigatus* to respond to antifungals such as amphotericin B and decreases the susceptibility towards these kind of drugs. In general, activated EneA by drugs or metabolites from streptomycetes, enhances the expression of genes within the neosartoricin/fumicyclin cluster. The produced metabolite inhibits the growth of microorganisms such as streptomycetes in the soil and impairs the immune response in the human host.

Previously, it was shown that direct contact of the fungus with the soil bacterium *S. rapamycinicus* results in the activation of the neosartoricin/fumicycline cluster^44^. In this study, we were able to show that the supernatant alone without bacteria is sufficient to induce the expression of the neosartoricin/fumicycline cluster in dependency of EneA (Fig. 4). This indicates that the activation of this SM cluster can occur by cell-cell contact and/or by specific metabolites of the bacterium (Fig. 6). Although the supernatant from an overnight culture of *S. noursei* does not contain nystatin, it can induce the expression of *eneA* and *nscA*. Therefore, it appears that *A. fumigatus* has the ability to respond to streptomycetes even before they can activate their defence mechanisms by producing polyenes.

It appears that EneA is an important factor for survival in the soil in the natural habitat of *A. fumigatus*, as it enables the fungus to compete with streptomycetes through the production of the antimicrobial agent neosartoricin^44^. Moreover, this response system can also react on polyenes like amphotericin B, which are used to treat fungal infection (Fig. 6). Activation of the *eneA* system enhances the production of neosartoricin (Fig. 6), which in turn impairs the immune response^38^. This presumably enables the fungus to better adapt within the host. Moreover, prolonged amphotericin B treatment, as administered to patients with chronic aspergillosis^48^, might lead to an increased accumulation of EneA, resulting in the activation of additional SM clusters with negative effects for the host.

In summary, in this work we analyzed a transcription factor called EneA, which is part of the polyene response system and acts as an enhancer of neosartoricin production. Moreover, EneA plays an important role in the regulation of other secondary metabolite cluster when it is overexpressed. The metabolites produced appear to protect the fungus against soil-dwelling bacteria and may negatively influence the immune response in aspergillosis patients^49^, which could increase the fungus’s viability within the host.

## Methods and materials

### Strains, media and growth conditions

*Escherichia coli* DH5α was used for routine cloning experiments and cultivated in LB medium (1% Bacto-tryptone, 0.5% yeast extract, 1% NaCl, pH 7.5) at 37 °C. Ampicillin was added at a final concentration of 100 µg/ml for selection.

*S. noursei* (DSM 40635) was obtained from Deutsche Sammlung von Mikroorganismen und Zellkulturen GmbH (DSMZ) and used for co-incubation experiments. The strain was grown in GYM medium (4 g/l glucose, 4 g/l yeast extract, 10 g/l malt extract, pH = 7.2). For solid medium, 2% (w/v) agar and 2 g/l CaCO_3_ were added. *S. noursei* cultures were incubated at 30 °C.

*A. fumigatus* was cultivated in minimal medium (MM) consisting of 1% glucose, 10 mM NaNO_3_ and 20 ml/l salt solution (348 mM KCl, 105 mM MgSO_4_, 558 mM KH_2_PO_4_, 50 ml/l trace elements^50^), adjusted to pH 6.5 with NaOH, unless stated otherwise. For solid media 2% agar was added. For selection pyrithiamine in a final concentration of 150 ng/ml or phleomycine in a final concentration of 20 µg/ml was used.

### Transformation procedures

*E. coli *transformation was performed according to Hanahan et al.^51^. Briefly, competent *E. coli* cells were incubated with DNA on ice for 30 min, followed by heat shock at 42 °C for 50 s. LB medium was then added, and 200 µl of the cell suspension was plated onto selective agar plates. Plates were incubated overnight at 37 °C.

*A. fumigatus* transformation was carried out following the protocol by Käfer^52^. Therefore, mycelium from a submerged overnight culture was incubated with VinoTaste Pro (novozymes, Bagsvaerd, Denmark) for 90 min at 30 °C to generate protoplasts. After adding DNA, protoplast fusion was induced using PEG4000 (10 mM Tris pH 7.5, 50 mM CaCl2, 60% PEG4000) and subsequently plated on selective medium.

### Manipulation of nucleic acid and purification

PCR was performed using Phusion polymerase (Thermo Fisher Scientific, Waltham, USA) according to the manufacturer’s instructions. Plasmid DNA was isolated using the “NucleoSpin Plasmid” kit (Macherey-Nagel, Düren, Germany) and DNA fragments were purified from agarose gels using the “NucleoSpin Gel and PCR Clean-up Kit“ (Macherey-Nagel, Düren, Germany). DNA isolation was carried out according to the protocol of Kolar et al.^53^. with one modification: Instead of phenol/chlorophorm, 8 M potassium acetate was used for precipitation. The “Geneart^®^ Seamless Cloning and Assembly kit” (Thermo Fisher Scientific, Waltham, USA) was used for plasmid construction and cloning steps. Sequencing of plasmids and PCR products was conducted by Seqlab Göttingen/Microsynth AG.

### Southern hybridization

Southern experiments were performed according to the method described by Southern et al.^54^. Therefore, 20 µg of DNA was digested overnight and afterwards separated *via* agarose gel electrophoresis. The gel was subsequently depurinated with 0.25 M HCl, denatured with 0.5 M NaOH and neutralized with 1.5 M NaCl/0.5 M Tris (pH 7.4). The DNA was transferred to an Amersham Hybond-N Nylon membrane (GE Healthcare Life Technologies, Little Chalfont, UK) and cross-linked. For probe labelling the “AlkPhos Direct Labeling Module” (GE Healthcare Life Technologies, Little Chalfont, UK) was used according to manufacturer’s instructions.

### Plasmid construction

The deletion construct of the *eneA* gene was performed as follows. Primers zcf63-6/zcf63-7 were used to amplify the 5’UTR of the *zcf63* coding sequence to receive a fragment with the size of 1.4 kb. This fragment was integrated via *Swa*I in the vector pChS314^55^. Afterwards a 1.1 kb fragment of the 3’UTR, was amplified using the primers zcf63-8/zcf63-9 and integrated via *Pml*I, resulted in the plasmid pChS248.

For construction of the *eneA* overexpression plasmid, primers zcf63-14/zcf63-15 were used to amplify the *eneA* gene (1.9 kb). The fragment was integrated via *Pme*I in the plasmid pME4313^56^ which contains a nitrate promoter for overexpression and a *H2A* termination region. The plasmid was named pChS388.

To generate the complementation plasmid pChS544, the *zcf63* gene was amplified using primers zcf63-10/zcf63-22. The *trpC* termination region was amplified from plasmid pChS399^10^ using primers trpCt-4/trpCt-8, yielding a 0.7 kb fragment had. Both fragments were integrated via *Swa*I into pChS248.

The *nscR* deletion plasmid was constructed as follows. The 5’UTR was amplified using primers zcf193-5 and zcf193-6 to receive a fragment of 1.3 kb and integrated via *Swa*I in the vector pChS314. For amplifying the 3’UTR the primers zcf193-7 and zcf193-8 were used to get a fragment of 1.2 kb. The fragment was integrated via *Pml*I in pChS314, which already contains the 5’UTR. The received vector was named pChS323.

The *nscA* deletion plasmid was constructed in the same way. Therefore, the primers pks11-5 and pks11-6 were used to amplify the 5’UTR and the primers pks11-7 and pks11-8 to get the 3’UTR. The received plasmid was named pChS318.

Unless stated otherwise, genomic DNA of the AfS35 was used as template. All plasmids and primers used are listed in supplementary Tables S10 and S11.

### Strain construction

The plasmid pChS248 was digested with *Pme*I and the resulting 7.9 kb fragment was integrated via homologous recombination in the AfS35 strain^57^ to receive the *zcf63* deletion strain. The received strain was named ACS289. For complementation, plasmid pChS544 was digested with *Pme*I and the 10.4 kb fragment was then integrated in the *eneA* deletion strain via homologous recombination to get ACS517. To construct the *nscR* deletion strain, plasmid pChS323 was digested with *Pme*I and the received 7.8 kb fragment was integrated in the *Tet-eneA* strain (ACS73) via homologous recombination to get ACS523. The *nscA* deletion strain was constructed by digesting the plasmid pChS318 with *Pme*I. The 7.3 kb fragment was then integrated in the *Tet-eneA* strain (ACS73) via homologous recombination. The received strain was named ACS333. Transformants were selected on minimal medium containing pyrithiamine. Marker recycling was performed as described previously^10,58^ by streaking transformants onto xylose containing medium. Correct genomic integration was confirmed by Southern hybridization.

The *eneA* overexpression strain was received by ectopic integration of the plasmid pChS388. Selection was performed on phleomycin-containing plates, and the resulting strain was designated ACS426. PCR was carried out for validation. All constructed and utilized strains are listed in supplementary Table S12.

### RNA isolation and qPCR experiments

*A. fumigatus* was inoculated with 2 × 10^6^ spores/ml in liquid minimal medium and incubated overnight at 37 °C. Cultures were incubated for an additional 4 h in presence or absence of amphotericin B in a final concentration of 0.65 µg/ml as mentioned in previous work^10^. For overexpression overnight cultures were incubated for additional 4 h with doxycycline in a final concentration of 50 µg/ml.

Total RNA was isolated with the “NucleoSpin RNA Plant Kit” (Macherey-Nagel, Düren, Germany) according to manufacturer’s instructions. cDNA synthesis was carried out using the “RevertAid First Strand cDNA Synthesis Kit” (Thermo Fisher Scientific, Waltham, USA). qRT PCR experiments were performed in a Bio-Rad CFX Connect Real-Time System or Bio-Rad CFX Duet Real-Time PCR System (Bio-Rad, Hercules, USA) using the “MESA GREEN qPCR kit for SYBR Assay” (Eurogentec, Lüttich, Belgium) according to the user’s manual. *H2A* and the *eIF2B* were used as reference genes. Primers used for qPCR can be found in supplementary Table S11. Statistical analysis was carried out using ANOVA for multiple comparison. If only two groups were compared the Student’s t-test was performed. Differences were considered significant with a p-value ≤ 0.05.

### Genome-wide transcriptional analysis (RNA-seq)

*A. fumigatus* was inoculated with 2 × 10^6^ spores/ml in submerged minimal medium and incubated overnight. Received mycelium were treated for additional 4 h with doxycycline (50 µg/ml final concentration). Total RNA was isolated as described above. Three biological replicates were prepared for each condition.

RNA sequencing was performed as described by Thieme et al.^59^. at the Core Unit, the Transcriptome and Genome Analysis Laboratory, University Medical Center Göttingen. For library preparation, 800 ng of RNA was used with the TruSeq Stranded Total RNA Sample Prep Kit from Illumina. (Cat. No. RS-122–2201). The library size and the quality was carried out with a Fragment Analyzer. The Promega’s QuantiFluor dsDNA System was used to determine the quantity of the constructed library. Sequencing was performed on an Illumina HighSeq-4000 platform (SR 50 bp; >30 Mio reads/sample). Raw reads were analyzed for quality control using the FastQC tool (Version 0.12.0). Read alignment was performed using HISAT2 software (Version 2.1.0) with *Aspergillus fumigatus* reference genome Af293 (Aspergillus_fumigatus.ASM265v1.42). Input parameters were “paired” for the library and “unstranded” for the strand information. Gene expression levels were quantified using the feature counts software (Version 1.6.3). The adjusted p-value of < 0.05 was used for significance. Functional category classification was performed using FunCat^35^.

### Extraction of secondary metabolites

Approx. 10^4^ freshly harvested spores were spotted onto minimal medium and incubated for seven days at 37 °C. Afterwards four pieces of 1.5 cm was punched out, transferred into a syringe, and extruded into a 50 ml Falcon tube. A mixture of water/ethyl acetate (ratio 1:1) was added to the shredded agar and incubated at RT under shaking conditions overnight. The following day, samples were centrifuged for five minutes at 2500 rpm, and the upper organic phase was transferred to a glass vial. The dried extract was used for further experiments.

For SM extraction used for the halo and the growth inhibition assays, 10^5^ spores were streaked onto minimal medium and incubated for seven days at 37 °C. Afterwards, 16 pieces each of 1.5 cm diameter were punched out, and extraction was performed as described above.

### Analysis of secondary metabolites

Extracts were prepared as described by Liu et al.^3^. Briefly, dried extracts were disssolved in 500 µl of acetonitrile/H2O (ratio1:1) and centrifuged for 10 min at 4 °C. Subsequently, 400 µl of the supernatant were transferred to a LC-MS vial. For LC-MS analysis a Q-Exactive™ Focus orbitrap mass spectrometer coupled to a Dionex Ultimate 3000 HPLC (Thermo Fisher Scientific, Waltham, USA) and equipped with a DAD 3000 diode array detector and a Corona Veo RS charged aerosol Detector was used. HPLC was carried out using a Acclaim™ 120, C^18^, 5 μm, 120 Å, 4.6 × 100 mm. 5 µl of each sample was injected for analysis. Running phase was set as a linear gradient from 5 to 95% (v/v) acetonitrile/0.1 formic acid in 20 min, plus 10 min with 95% (v/v) acetonitrile/0.1 formic acid) with a flow rate of 0.8 ml/min at 30 °C in addition. The measurements were performed in positive and negative modes with a mass range of 70–1050 m/z. For data analysis the software FreeStyle™ 1.4 (Thermo Fisher Scientific, Waltham, USA) was used.

### Extraction of secondary metabolites from *S. noursei* and *A. fumigatus* from submerged cultures

For submerged cultures, 100 ml of minimal medium was inoculated with approximately 2 × 10^8^ spores of *A. fumigatus* AfS35 and incubated overnight at 37 °C. *S. noursei* harvested from GYM plates (one plate per culture) were used for inoculation of 100 ml of liquid GYM medium and incubated overnight at 30 °C.

For each monoculture, 100 ml of culture broth was extracted with ethyl acetate at a 1:1 (v/v) ratio. For co-culture experiments, 100 ml of each monoculture was combined in a new flask and incubated for an additional 4 h at 30 °C. A total of 200 ml of the mixed culture were used for SM extraction with ethyl acetate (ratio 1:1). LC-MS analysis was performed as described above. Nystatin (1 µg) was used as a reference standard.

### Dilution assays

Dilution spot-tests were carried out as described previously^60^. Briefly, *A. fumigatus* spores were harvested, and serially diluted from an initial concentration of 5 × 10^7^ spores/ml. Aliquots of 3 µl of each dilution step was spotted on medium with and without drug. Amphotericin B (final concentrations of 0.5 µg/ml, 0.75 µg/ml, 1 mg/ml, and 1.25 µg/ml) and nystatin (in a final concentration of 3 µg/ml) were used as drugs. For the *Tet*-overexpression strains, doxycycline was added in a final concentration of 50 µg/ml.

### Growth inhibition assay and halo test

Dried extracts obtained as described above were dissolved in 150 µl MeOH and 10 µl were finally added on a paper disc. The disc was placed at the center of a minimal medium plate containing 1 × 10^7^ embedded *A. fumigatus* spores and amphotericin B in a final concentration of 1.5 µg/ml. Plates were incubated for three days at 37 °C. Three independent experiments, each with three technical replicates, were performed. The size of the fungal colony ring was measured in pixel per area. Statistical analysis was carried out using the student’s t-test and significance was determined by a p-value ≤ 0.05.

For the halo assay, 100 µl of *S. noursei* spore suspension, received from a single plate, was streaked out on GYM medium. Paper discs containing the same concentration of SM extracts used for the *A. fumigatus* diffusion test were placed at the center of the plate. Plates were incubated two days at 30 °C. Three independent experiment were performed, each with three technical replicates.

### Co-incubation of *A. fumigatus* and *S. noursei*

*S. noursei* spores harvested from a single agar plate were inoculated into 100 ml of liquid GYM liquid medium and incubated at 30 °C overnight. *A. fumigatus* was inoculated with 2 × 10^6^ spores/ml in liquid minimal medium. Submerged cultures were incubated overnight at 37 °C. For co-cultivation 100 µl of the *S. noursei* overnight culture or 100 µl of the supernatant were added to the *A. fumigatus* mycelium and the mixture was incubated for additional 4 h. As control, 100 µl fresh GYM medium was added to *A. fumigagtus*. The mycelium was subsequently harvested and used for RNA extraction as described above.

## Supplementary Information

Below is the link to the electronic supplementary material.

Supplementary Material 1

Supplementary Material 2

Supplementary Material 3

Supplementary Material 4

Supplementary Material 5

Supplementary Material 6

Supplementary Material 7

## Data Availability

The data used in this study are provided in the main text or in the supplemental material. RNAseq raw data files were deposited with NCBI (Accession: PRJNA1257291).
