# Correction: EneA of *Aspergillus fumigatus* is a regulator of secondary metabolism and enhances *nscA* expression in presence of polyenes and *Streptomyces*

**DOI:** 10.1038/s41598-026-62198-8

**Published:** 2026-08-06

**Authors:** Oskar Bunz, Jennifer Gerke, Oliver Bader, Emmanouil Bastakis, Merle Aden, Kai Heimel, Ralf Bürgers, Gerhard H. Braus, Christoph Sasse

**Affiliations:** 1 Department of Prosthodontics, University Medical Center Goettingen, Göttingen, Germany; 2 Institute of Organic Chemistry, Leibniz University Hannover, Hannover, Germany; 3 Institute for Medical Microbiology and Virology, University Medical Center Goettingen, Göttingen, Germany; 4 Institute of Microbiology and Genetics, Department of Molecular Microbiology and Genetics and Goettingen Center for Molecular Biosciences (GZMB), University of Goettingen, Göttingen, Germany; 5 Institute of Microbiology and Genetics, Department of Microbial Cell Biology, Göttingen Center for Molecular Biosciences (GZMB), University of Goettingen, Göttingen, Germany

Correction to: *Scientific Reports* 10.1038/s41598-026-47215-0, published online 09 April 2026

The original version of this Article contained an error in Figure 5 where the labels “SM wt” and “SM OEeneA were swapped in panel (a). The original Figure 5 and accompanying legend appear below.

The original Article has been corrected.

**Fig. 5:**
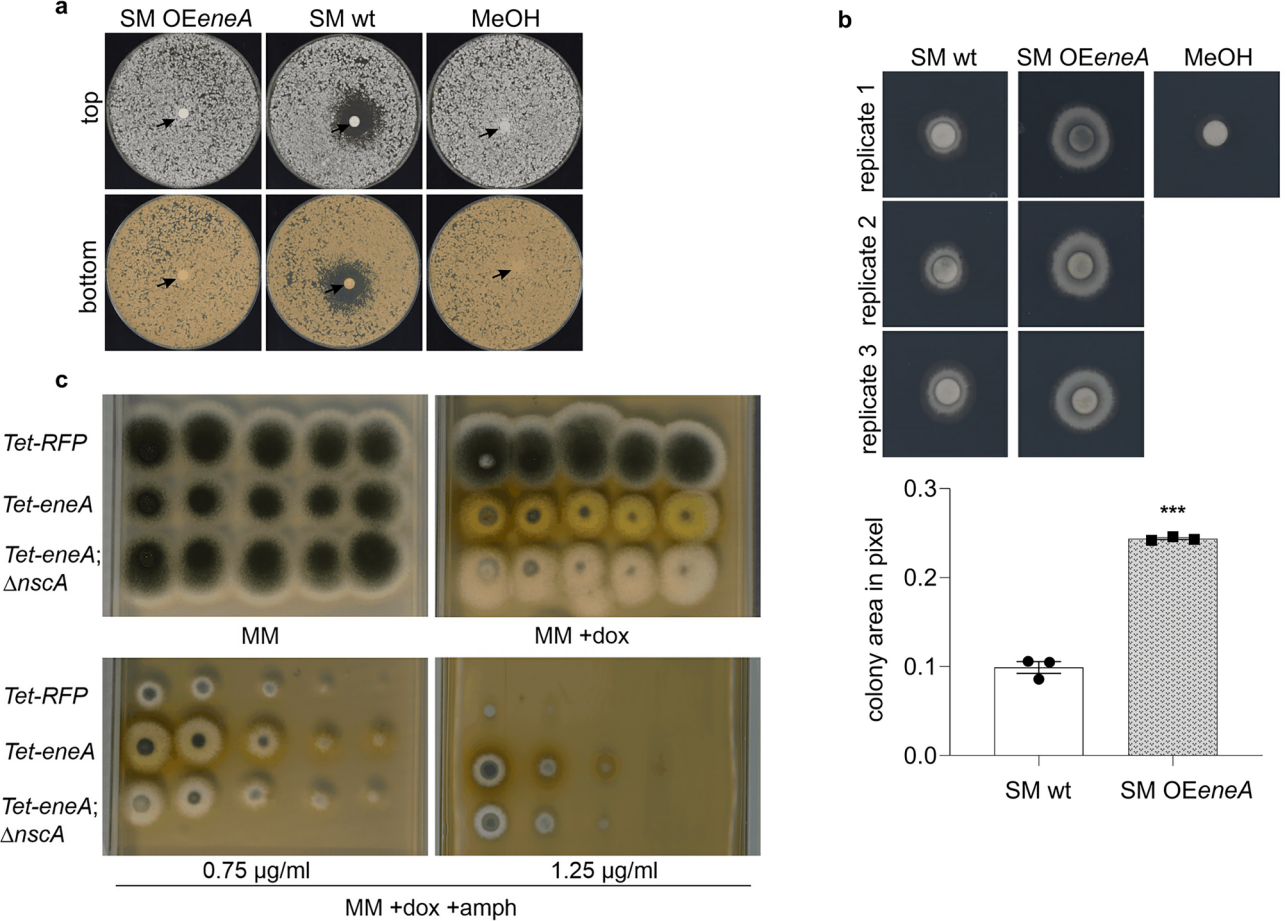
Secondary metabolites from the *A. fumigatus eneA* overexpression strain neutralize amphotericin B and inhibit the growth of the soil bacterium *S. noursei*. **(a)** Disc diffusion assays using extracts of the *OEeneA* strain and the wild type. *S. noursei* was inoculated on GMX plates in presence of fungal extracts (overexpression strain, wild type). Black arrows indicate the paper discs soaked with the two different extracts. As control a paper disc with methanol was used. Growth inhibition is indicated by a halo around the apper discs. (**b**) Plates containing embedded spores of the *(A) fumigatus* wild type and 1.5 µg/ml amphotericin B. In the middle of the plate, a paper disc was added which was soaked with extracts containing secondary metabolites (SM) from the *eneA* overexpression strain or from the wild type. As control, a paper disc containing methanol was used. Plates were incubated for three days at 37 °C. The growth area of the plates were determined and statistical analyzed using student’s t-test with *n* = 3. *** ≙ p-value ≤ 0.0005. Circles and squares represent the individual values of the replicates. Only the extract received from the overexpression strain increases significantly the growth of the fungus in presence of amphotericin B (**b**) and inhibits the growth of the bacterium indicated by a growth halo around the paper disc (**a**). The figure shows exemplary plates. All plates are shown in supplementary Fig. S3. Dilution spot test of the *Tet-prrA* strain and the *Tet-prrA*; Δ*nscA* strain. As reference, the *Tet-RFP* strain was used. Strains were spotted on minimnal medium (MM) minimal medium doxycycline (+ dox) or minimal medium with amphotericin B and doxycycline (+ dox +amph). Amphotericin B was used in a final concentration of 0.75 mg/ml and 1.25 µg/ml. Doxcycline was used in a final concentration of 50 µg/ml. Plates were incubated for three days at 37 °C (**c**). The absence of *nscA* does not increase the susceptibility to amphotericin B.

