# Supplementary material for: EneA of Aspergillus fumigatus is a regulator of secondary metabolism and enhances nscA expression in presence of polyenes and Streptomyces
Source: Sci Rep. 2026 Apr 9;16:12038. doi: 10.1038/s41598-026-47215-0 (PMC13068945; doi:10.1038/s41598-026-47215-0)
Supplement: Supplementary file 1 — Supplementary Material 1 [file 41598_2026_47215_MOESM1_ESM.pdf]

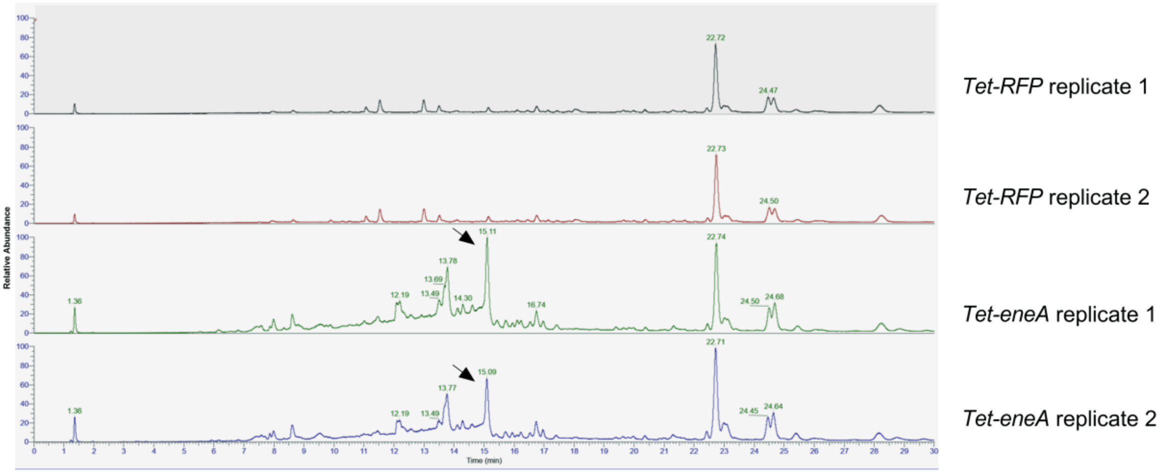

Fig. S1: Charged aerosol detector (CAD) chromatograms of secondary metabolites (SMs) of the *Tet-eneA* overexpression strain and the *Tet-RFP* strain. Conidia of the *Tet-eneA* strain and the *Tet-RFP* control strain of *A. fumigatus* were point-inoculated on minimal medium with 50 µg/ml doxycycline for seven days at 37°C. Extra- and intracellular metabolites were extracted and detected by LC-MS with a charged aerosol detector (CAD). Extracts from two independent plates of each strain were analysed. Black arrows indicate the peak of neosartoricin/fumicycline at retention time 15.11 and 15.09 respectively.

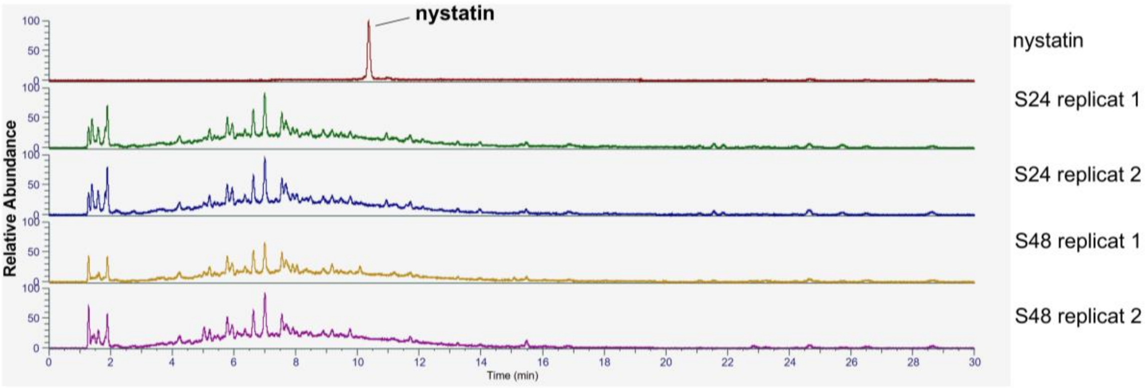

Fig. S2: Charged aerosol detector (CAD) chromatograms of secondary metabolites (SMs) from *S. noursei* submerged cultures. Secondary metabolites were extracted from submerged cultures of *S. noursei* after 24h (S24h) and 48h (S48h) incubation at 30°C in GYM medium. Two replicates were analyzed. Detection of metabolites was performed by LC-MS with a charged aerosol detector (CAD). 1 µg of pure nystatin was used as reference.

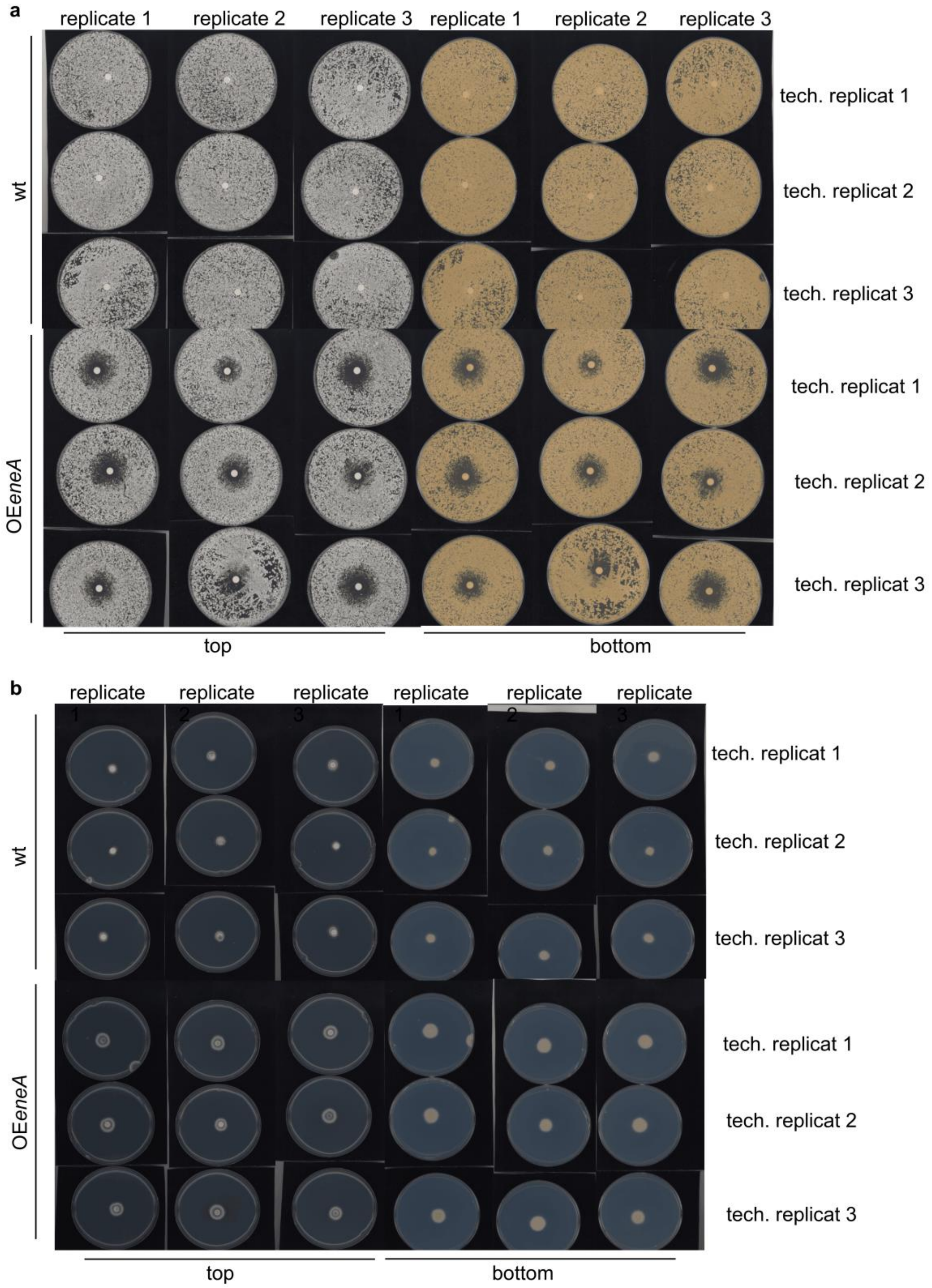

Fig. S3: Influence of secondary metabolites of an *ene*A overexpression strain on amphotericin B and *S. noursei*. (**a**) Influence of SM from wild type and *eneA* overexpression strain on the growth of *S. noursei*. A paper disc soaked with metabolites from the wt or the *OEeneA* overexpression strain were placed on plated spreaded with *S. noursei*. A halo around the paper discs indicates inhibited growth of the bacterium. (**b**) Plates containing embedded spores of the *A. fumigatus* and contain 1.5 µg/ml amphotericin B. In the middle of the plate, a paper disc was added which was soaked with extracts containing secondary metabolites (SM) from the *eneA* overexpression strain as well as from the wild type. Plates were incubated for three days at 37°C. Three independent replicates with three technical replicates were performed for each experiment (see supplementary Fig. S3).
